# How can physical enrichment of school playgrounds improve movement behaviours and developmental outcomes in children and adolescents? A systematic review with meta-analysis

**DOI:** 10.1186/s12966-025-01856-y

**Published:** 2025-11-22

**Authors:** Luca Oppici, Katrine N. Aadland, Eivind Aadland, Minghui Li, James R. Rudd

**Affiliations:** 1 The Department of Teacher Education and Outdoor Studies, Norwegian School of Sport Sciences, Oslo, 0863 Norway; 2 Monash University European Research Foundation ETS, Prato, Italy; 3 Department of Sport, Food and Natural Sciences, Faculty of Education, Arts and Sports, Western Norway University of Applied Sciences, Campus Sogndal, Sogndal, 6856 Norway; 4 Faculty of Sports Science, Ningbo University, Ningbo, China

**Keywords:** Enriched environment, Environmental enrichment, Physical activity, Motor development, Children and youth, Social skill, Social interaction, School

## Abstract

**Objective:**

This study reviewed (1) the effects of physical enrichment of (pre-)school playgrounds on children’s and adolescents’ movement behaviours and their cognitive, psychological, and social development, and (2) how enrichment characteristics influenced these outcomes.

**Methods:**

A systematic review with meta-analysis was conducted following the PRISMA guidelines. We searched 7 databases (SPORTDiscus, CINAHL, MEDLINE, APA PsycINFO, Cochrane Library, Web of Science, and Scopus) and two reviewers independently screened studies for eligibility. Effect sizes of interventions were calculated using standardized mean differences (Hedges’ g) and tested using random-effects models. Meta-regression was used to explore heterogeneous findings. Narrative syntheses were conducted when meta-analysis was not possible.

**Results:**

Twenty-eight studies and 19,753 participants were included. Three studies had low risk of bias, 11 studies had high risk of bias, while 14 studies had some concerns. The meta-analyses for movement behaviours showed significant increases in vigorous physical activity (VPA, *n* = 7 studies, g = 0.17, 95% Confidence Interval (CI): 0.06, 0.27), moderate-to-vigorous physical activity (MVPA, *n* = 17, g = 0.17, CI: 0.03, 0.30), and steps per minute (*n* = 3, g = 0.81, CI: 0.03, 1.59) for enrichment interventions relative to control conditions. The results for MVPA had large heterogeneity and were only significant short-term. Line markings alone or in combination with physical structures (e.g., climbing walls) increased MVPA, while involving end-users decreased MVPA. Line markings alone or in combination with physical structures or equipment increased VPA. There was no effect of enrichment for sedentary behaviour, low or moderate intensity physical activity. The results for cognitive (attention restoration and executive functioning), psychological (wellbeing and quality of life), and social (interactions, orientations, and bullying) development were mixed.

**Conclusion:**

Our findings show that studies on physical enrichment had low methodological quality and findings had high heterogeneity. Line markings in isolation or combined with other enrichment strategies might increase levels of VPA and MVPA in children and adolescents. Evidence on cognitive, psychological and social development is inconclusive due to few and weak studies for these outcomes. We provide future directions for research and practice by drawing from the environmental enrichment paradigm.

**Trial registration:**

PROSPERO registration number CRD42022364392.

**Supplementary Information:**

The online version contains supplementary material available at 10.1186/s12966-025-01856-y.

## Introduction

 Internationally, governments, health professionals, and exercise practitioners are grappling with the increasing threat posed by physical inactivity, which leads to poorer health outcomes, reduced life expectancy, and substantial economic burdens [1, 2]. Schools are a critical setting for physical activity promotion due to their near-universal reach, daily structure, and infrastructure that supports movement [3]. Globally, over 770 million children are enrolled in primary and secondary education [4, 5], positioning schools as uniquely placed to implement equitable and large-scale health-promoting interventions. Schools are likely to include playful environments, such as playgrounds where physical activity and social interactions are central to the educational experience. Particularly during recess and lunch breaks, schools offer many opportunities for play, movement, exploration, and social engagement. An important challenge is how to enhance both the quality and quantity of opportunities for physical activity and social interactions in these environments. Physical enrichment of school playgrounds is a promising strategy.

Physical enrichment entails modifications of the physical aspects of an environment with the aim of creating enhanced opportunities for movement, cognitive and social behaviours, for which different strategies can be adopted to physically enrich school playgrounds. The surface of playgrounds can be painted with lines and shapes of different colours to create areas for movement and play, encouraging individuals to be physically active in a variety of behaviours [6, 7]. For instance, a playground can be divided in zones with each zone inviting specific behaviours (e.g., walking or running), and paintings can create themes of play that match the children’s interest and preference. Another strategy is to restructure the physical environment [8]. For instance, the ground can be changed to natural or artificial grass, and new facilities can be built to promote active behaviours, such as parkour area, fitness and skate facilities and climbing walls. Lastly, equipment can be added to a playground, including racquets, balls, rings, and car tyres, inviting children to play, move, socialize, and have fun [9].

Previous literature has primarily focused on the effect of physical enrichment on physical activity levels [10–12]; however, physical enrichment may also influence a range of motor, psychological, cognitive, and social development [9]. Physical enrichment directly aims to increase movement behaviours, which include but should not be restricted to physical activity level. It should also include motor skills, as enrichment invites children to perform a variety of movement activities (e.g., jumping, catching and throwing) and exploration with their body [13]. Further, considering the known link between physical activity and cognition [14, 15], if enrichment interventions increase physical activity, they could also lead to cognitive benefits, such as improvement of executive functioning and attention span. Enrichment may also directly tap into other mechanisms, such as fostering social interactions and improving wellbeing as a result of participating in fun activities. In short, physical enrichment has the potential to influence movement behaviours, as well as psychological, cognitive and social development.

Previous reviews has provided conflicting results on the effect of physical enrichment on children’s and adolescents’ physical activity. Parrish et al. [11] found inconclusive evidence, Escalante et al. [12] found a positive effect of playground markings plus physical structures, and Broekhuizen et al. [9] found a positive effect of equipment and inconclusive evidence for the other strategies. Broekhuizen et al. [9] looked beyond the effect on physical activity, but found inconclusive evidence for cognitive and social outcomes. As such, it is currently unclear how physical enrichment of school playgrounds influence behaviours and development of children and adolescents.

The aim of this study was to review current evidence on the effect of physical enrichment of (pre-)school playground on children’s and adolescents’ movement behaviours, and their cognitive, psychological, and social development 1. Further, we aimed to examine the different strategies of physical enrichment and their effects on these outcomes. Lastly, we aimed to explore how physical enrichment was implemented and supported by staff and key stakeholders. In this review, movement behaviours refer to waking behaviours involving bodily movement, including physical activity of various intensities (of light, moderate, moderate-to-vigorous, and vigorous intensity) and step count, sedentary time, and motor competence. Sleep behaviours were outside the scope of this review.

## Methods

The current study follows the guidelines proposed by the 2020 Preferred Reporting Items for Systematic Reviews and Meta-Analyses [PRISMA; 16], and it was registered in PROSPERO (reference number: CRD42022364392) prior to starting data collection. The PRISMA checklist is provided in the Supplementary Materials.

### Eligibility criteria

The inclusion and exclusion criteria for this study were set using the PICOS statement (for details, please see the Supplementary Materials):

Inclusion: P(population): 2- to 18-year-old typically developing individuals attending education in early years and/or school environments; I(intervention): physical enrichment of playful environments in the school context, such as line markings and the addition of equipment, as the main intervention strategy; C(comparator): Any form of control group with no physical enrichment, including usual care or wait list control; O(outcome): Children’s physical (e.g., movement behaviours including total physical activity, intensity-specific physical activity and sedentary time, motor competence etc.), cognitive, psychological/affective, and/or social development; S(study design): randomized controlled trial (RCT), cluster-RCT, non-randomized controlled trial.

Exclusion: P(population): Participants beyond the age range or from specific populations; I(intervention): Interventions conducted in settings beyond school, and multi-component interventions with a strong role of the teacher, such as enrichment combined with a new pedagogical approach to increase students’ activity; C(comparator): Interventions compared two active intervention arms without a comparison/control group; O(outcome): Other outcomes; S(study design): Systematic review, observational studies, and descriptive studies with no data.

Although being reported in the PROSPERO registration, it was not possible to include articles published in multiple languages (i.e., including English, Chinese, and Scandinavian), due to large differences in the search systems and the lack of advanced search function across the different countries and languages, which prevented a consistent and efficient search. As such, the search was restricted to peer-reviewed articles in English.

### Information sources and search strategy

A comprehensive literature search was conducted in the following databases: SPORTDiscus, CINAHL, MEDLINE (through Pubmed), APA PsycINFO, Cochrane Library, Web of Science, and Scopus. The search was initially performed on the 23rd of September 2022, and it was updated on the 10th of June 2025. The references of the studies included in the review and relevant review articles were screened as a means of identifying additional relevant studies.

Search terms combined the domains of “setting” (school* or school-based or kindergarten or “early learning center” or childcare or “childcare center” or preschool or pre-school or college), “population” (child* or adolescen* or youth or “young people” or teen* or student* or pupil* or infant or toddler), and “intervention” (enriching or modification or manipulation or upgrade or rebuilding or renovate or adapting or variation greenness or marking or equipment or material or environment or playground or schoolyard or “playful environment”). Search strings and syntax were tailored to the characteristics of each database (for the full list of search strings, see the Supplementary Materials). The search had no restrictions on publication dates.

### Selection process

All records identified through the searches were exported into Endnote X9 software (Clarivate, Philadelphia, USA) by one researcher (ML). Duplicates were removed automatically and checked manually for unrecognized duplicates by one researcher (ML). Two authors (ML and KNA) independently screened titles and abstracts first, and then screened the full-text articles to determine final eligibility. Further, the two authors independently screened the reference lists of the included studies to identify further relevant studies. Disagreements between the two reviewers were discussed and resolved together with two other authors (JR and LO) who were independent to the search process.

### Data extraction and synthesis

The following data was extracted from each study: general study information (author, year, location, study design, target population), sample characteristics (sample size, age range, gender %), types and details of enrichment (strategies used, teachers’ role, etc.), intervention duration, and outcomes measures. To better characterize the enrichment design of the included studies, the extracted data was then coded using 5 umbrella themes derived from recent developments in environmental enrichment research (see in the discussion how this experimental paradigm can advance current enrichment approaches) [17]. The 5 themes encapsulate: i) complexity, the type of strategies used to increase complexity in the playground (adding objects [equipment and structures] and/or modifying spatial areas [line markings], ii) variety, whether the intervention encouraged a variety of activities and behaviours, iii) novelty, whether the strategy included changes throughout the intervention to constantly provide novel activities, iv) targeting needs, which children’s and youths’ needs the intervention targeted and whether the end-users’ voice was considered in the intervention design, v) and scaffolding, whether the intervention was implemented progressively and incrementally and how. Two authors (ML and KNA) split the included studies in half; each author extracted the data from half of the studies; they exchanged their results and performed an accuracy check.

### Risk of bias assessment

The Risk of Bias (RoB) was assessed for each eligible study by two authors independently (ML and KNA) using the Excel spreadsheets available at https://www.riskofbias.info/welcome. The authors crosschecked their results and resolved any disagreements together with two additional authors (JR and LO). Tools developed by the Cochrane group were used for assessing the risk of bias [18]. The RoB 2 tool for cluster-randomized trial (RoB 2 CRT) was used for assessing studies with a cluster-randomized design. This tool comprises 6 bias domains: (1) randomization process, (1b) timing of identification or recruitment of participants, (2) deviations from the intended interventions, (3) missing outcome data, (4) measurement of the outcome, and (5) selection of the reported results. The ROBINS-I (Risk Of Bias In Non-randomised Studies – of Interventions) tool was used for evaluating the risk of bias in non-randomized studies [19]. This tool is comprised of 7 bias domains: (1) confounding, selection of participants into the study, (2) classification of interventions, (3) deviations from the intended interventions, (4) missing data, (5) measurement of outcomes and (6) selection of the reported results. Both tools contain signaling questions to facilitate the assessment of the potential bias in each domain. In the RoB 2 CRT, there are 3 possible outcomes in each domain—low risk, some concerns, and high risk— while there are 5 possible outcomes in the ROBINS-I tool—low risk, moderate risk, serious risk, critical risk and no information. To evaluate and synthesize the studies included in the review parsimoniously, we decided to adjust the rating of the ROBINS-I tool to the rating of the RoB 2 CRT, consistent with previously published literature [20]. As such, low risk remained unchanged, moderate risk and no information were classified as some concerns, whereas serious risk and critical risk were classified as high risk. An overall risk of bias, corresponding to the highest risk across domains, was calculated for each study (i.e., if the risk was *some concerns* in one domain only but *low* in all other domains, the overall risk was *some concerns*). The results of the risk of bias assessment are presented using the traffic light system: green (low risk), yellow (some concerns), and red (high risk) [21].

### Synthesis methods

This review included several outcomes. A meta-analytic integration of the results was performed for the outcomes that originated from at least 2 studies and with at least 200 participants. For the outcomes not reaching these thresholds, a narrative synthesis was conducted.

The Comprehensive Meta-Analysis software, version 4 (Biostat Inc., Englewood, USA) was used to run the meta-analyses. Standardized mean differences (Hedge’s g [SMD]) and their 95% Confidence Interval (CI) were computed between the experimental and control groups based on the means and Standard Deviations (SD) of the outcome of interest. These data were directly extracted from the articles, and, when not publicly available, the corresponding author was contacted to obtain the data. When a study provided outcomes at different time-points (e.g., post-intervention and follow-up), the means and SDs were initially averaged across the time-points. Due to high heterogeneity, studies were then grouped based on their assessment point, creating a group of studies with assessment point below 12 months and a group of studies with assessment point equal to or above 12 months (12 months was the median assessment time point). A meta-regression was performed to better understand heterogeneity in studies and examine the moderator role of the different enrichment strategies, using the 5 themes described under data extraction. The random-effects model was employed for the analysis. The studies in the analysis are assumed to be a random sample from a universe of potential studies, and this analysis will be used to make an inference to that universe [22]. The SMDs of individual studies and the pooled effect were presented using a forest plot. SMDs of ≥ 0.2, 0.5, and 0.8 were interpreted as small, moderate, and large, respectively [23]. The statistical significance threshold was set to alpha = 0.05.

Heterogeneity (i.e., the dispersion of the effect sizes across studies) was evaluated using the prediction interval [24–26], calculated using the Comprehensive Meta-Analysis. Importantly, the confidence interval of the SMD represents the precision of the estimate of the mean, while the prediction interval indicates how disperse the true effect sizes are across the populations [27]. I^2^ (percentage of variance in observed effects that reflect variance in true effects rather than sampling error), Tau^2^ (variance of true effects), and Tau (standard deviation of true effects) were also reported for completeness. To evaluate the impact of individual studies on the pooled effect, a sensitive analysis was computed using the “remove one study” feature of the Comprehensive Meta-Analysis, which computes the effect removing each study individually (large variation in the effect would indicate the impact of a specific study). Lastly, potential publication bias was assessed using the Trim-and-Fill method on funnel plot asymmetry [28].

### Assessment of the quality of the evidence

The level of evidence for each of the outcomes of interest was assessed using the Grading of Recommendations Assessment, Development and Evaluation (GRADE) approach [29]. Each outcome was evaluated individually, and the level of evidence was rated as high, moderate, low, or very low based on 9 assessment criteria. The study design serves as the starting point: RCTs are rated high and non-RCTs are rated low. Then, the initial rating can be downgraded based on risk of bias, inconsistency, indirectness, imprecision, and publication bias, and/or upgraded based on the magnitude of the effect size, dose response, and no plausible confounding [30]. Two authors (ML and LO) graded the level of evidence independently, cross-checked their results and resolved any conflicts.

## Results

**Fig. 1:**
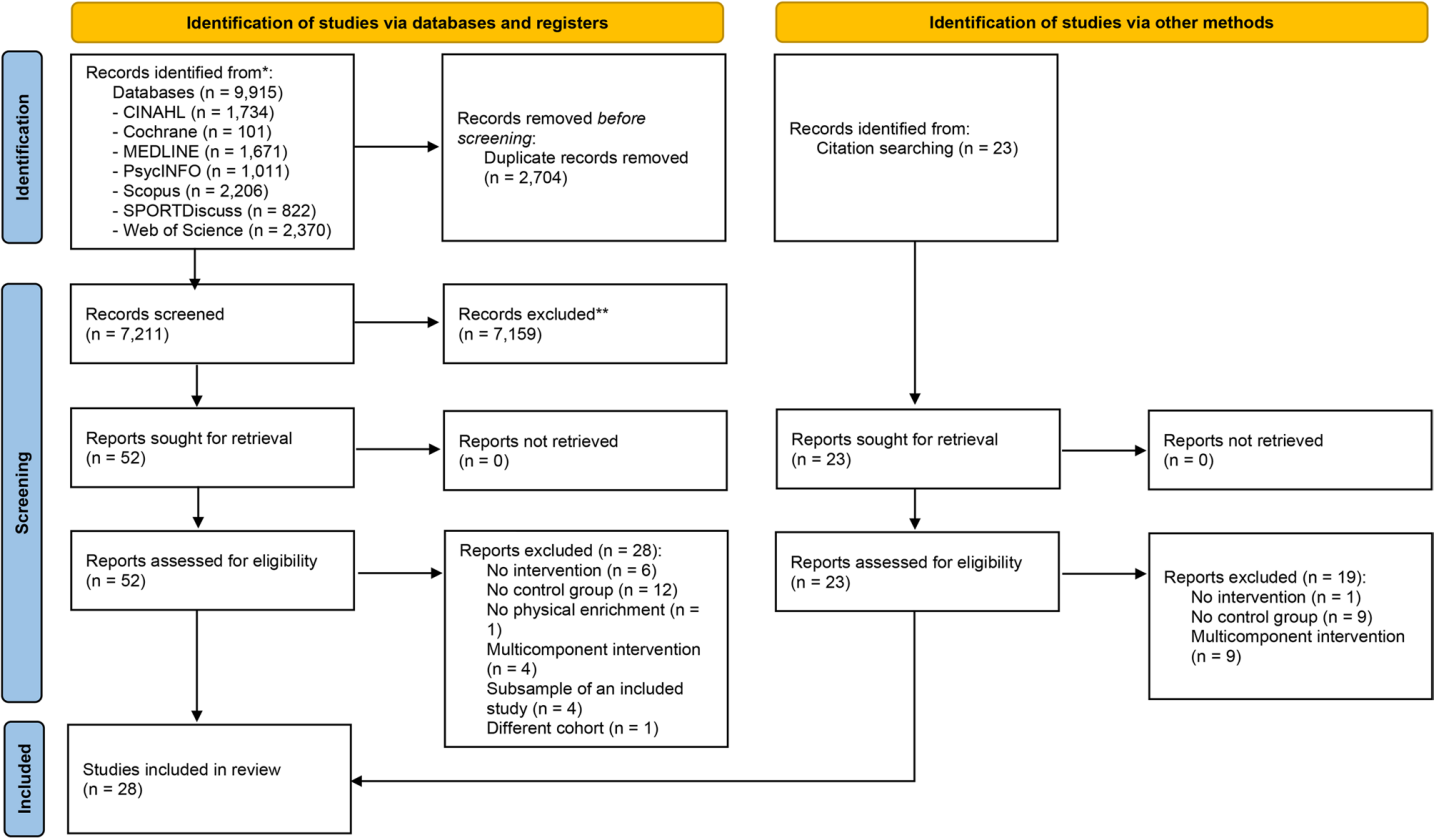
PRISMA flow diagram of the screening process

The initial search through the databases identified 9915 studies (CINAHL, *n* = 1734; Cochrane, *n* = 101; MEDLINE, *n* = 1671; PsycINFO, *n* = 1011; Scopus, *n* = 2206; SPORTDiscuss, *n* = 822; Web of Science, *n* = 2370). After the removal of 2704 duplicates, 7211 studies were excluded based on their title and abstract. The full text of 52 studies was read, of which 24 were included in the review. The search through the studies’ reference lists resulted in 4 additional studies being included. As such, a total of 28 studies were included (Fig. 1). Four additional studies met the inclusion criteria [31–34] but were subsamples of other included studies and, thus, were not included in the review. Three studies were reported in 2 articles each [7, 35–39], and both articles from each study were included because they reported different outcomes. The characteristics of these studies were presented in combination, but their risk of bias and outcomes were analysed separately in separate analyses. As such, 28 articles, reporting 25 studies, were included in the review.

### Study characteristics

The characteristics of the included studies are presented in detail in Table 1. The included studies involved a total of 19,753 participants, with sample sizes ranging from 60 to 6500 participants (mean = 790 [SD = 1323], median = 338). The participants’ mean age ranged from 2 to 15 years, and the percentage of boys and girls was balanced across the studies (mean = 52% boys). Participants attended pre-school in 3 studies [42, 50, 53], primary school in 23 studies [6, 7, 35–41, 43–49, 51, 52, 54, 55, 57, 59], and secondary school in 3 studies [8, 56, 60].

**Table 1:** Characteristics of the studies included in the review

Study, year, country	Design, and sample (experimental/total, age range, % boys)	Details of the enrichment intervention	Intervention duration	Outcome measures and instruments
**Primary Outcome: Movement behaviour**				
Baquet et al. [40], 2018, France	Cluster-RCT; primary school environment 3 schools (2 to the intervention group) 185/283 (6–11 years old; 50% boys)	Intervention group: Markings of the playground; even spacing of facilities (e.g., fun trails and dens, ladders) across the playground area; provision of play equipment (e.g., rackets, balls, huge dies chess, scarfs, and hockey sticks) Role of teachers: Supervision Control group: Business as usual	12 months	Time in SED, LPA, MVPA, measured with accelerometers during recess (break) periods at baseline, and 6 months and 12 months after baseline
Blaes et al. [41], 2013, France	Cluster-RCT; primary school environment 4 schools (2 to intervention group) 197/420 (6–11 years old; 51% boys)	Intervention group: Markings of the playground; even spacing of facilities (e.g., fun trails and dens, ladders) across the playground area; provision of play equipment (e.g., rackets, balls, huge dies chess, scarfs, and hockey sticks) Role of teachers: Not specified Control group: Business as usual	2 weeks	Time in SED, LPA, MPA, VPA, MVPA, measured with accelerometers during morning and afternoon recess at baseline and 2 weeks after baseline
Cardon et al. [42], 2009, Belgium	Cluster-RCT; Pre-school environment 40 pre-schools (30 to the intervention group) 472/634 at baseline (mean age 5 years; 52% boys)	Intervention groups: (1) provision of play equipment (different ball types, throwing discs, throwing rings, aiming rings, bean bags, hoops, jumping bags); (2) markings of the playground; (3) both play equipment and markings Role of teachers: Introduce the equipment and make it available during all recesses Control group: Business as usual	4–6 weeks	Time in SED, LPA, MPA, VPA, MVPA measured with accelerometer during recess time at baseline and 4–6 weeks after baseline
Christiansen et al. [8], 2017, Denmark	Cluster-RCT; Secondary school environment 14 schools (7 to the intervention group) 623/1348 (12–14 years old; 50% boys)	Intervention group: Upgrade of the outdoor area, including provision of unfixed equipment, markings, creation of areas for ball games, and design of a playspot (climbing, parkour, fitness and skate facilities) Role of teachers: Facilitate and motivate PA during recess Control group: Business as usual	6 months	Time in MVPA measured with accelerometers at baseline and at least 6 months after baseline
Engelen et al. [38], 2013, Australia Bundy et al. [37], 2017, Australia	Cluster-RCT; primary school environment 12 schools (6 to the intervention group) 113/226 (5–7 years old; 54% boys)	Intervention group: Provision of loose, primarily recycled, materials (i.e., car tires, milk crates, and fabric) Role of teachers: No defined role. Teachers and parents participated in a 2-hour group intervention to discuss experiences on free play Control group: Business as usual	13 weeks	Primary outcome: Total counts and time in SED, LPA, MVPA measured with accelerometers during break times and whole school day at baseline and 13 weeks after baseline Secondary outcomes: Social interactions measured with video recordings at baseline and 13 weeks after baseline Children’s and teacher’s perceptions of social acceptance Parent and teacher reports on social skills
Farmer et al. [36], 2017, New Zealand Farmer et al. [35], 2017, New Zealand	Cluster-RCT; primary school environment 16 schools (8 to the intervention group) 422/840 (6–9 years old; 46% boys)	Intervention group: Redesign of playgrounds to encourage imaginative and independent free play by increasing opportunities for risk and challenge, reducing rules, and adding new playground components (e.g., loose parts) Role of teachers: Not defined Control group: Business as usual	2 years	Primary outcome: Counts per minute and time in MVPA measured with accelerometers during break times and whole day at baseline, and 1 year and 2 years after baseline Secondary outcome: Bullying reported by children, teachers, and parents using the PRAQ-R questionnaire
Hamer et al. [43], 2017, UK	Non-RCT; primary school environment 7 schools (5 to the intervention group) 169/231(mean age 8 years, 55% boys)	Intervention group: Playground reconstruction using AstroTurf game pitches, climbing frames, trampolines, monkey bars and outdoor gyms Role of teachers: Not defined Control group: Business as usual	1 year	Time in SED, LPA, MVPA measured with accelerometers during school and whole day at baseline and 1 year after baseline
Hyndman et al. [44], 2014, Australia	Non-RCT; primary school environment 2 schools (1 to the intervention group) 123/275 (5–12 years old; 54% boys)	Intervention group: Provision of moveable and recycled materials with no fixed purpose (e.g., milk crates, buckets, cardboard boxes, tyres, exercise mats), balls, hoops and skipping ropes Role of teachers: Usual supervision Control group: Business as usual	8 months	Primary outcome: PA measured with pedometers (number of steps) and SOPLAY at baseline, and 7 weeks and 8 months after baseline Secondary outcome: Quality of life using the Peds QL 4.0
Huberty et al. [45], 2014, US	Cluster-RCT; primary school environment 12 schools 180/371 (7–12 years old, 46% boys).	Intervention group: Provision of play equipment (basketballs, frisbees, beach balls, hula hoops, cones etc.) Role of teachers: No active role Control group: Business as usual	8–9 months	Time in SED, MVPA measured with accelerometers and SOPLAY tool during break times at baseline and 8–9 months after baseline
Janssen et al. [46], 2015, The Netherlands	Cluster-RCT; primary school environment 8 schools (4 to the intervention group) 1155/2310 (6–12 years old; 56% boys)	Intervention group: Restructuring of the playground by multi-coloured markings and the provision of play equipment (i.e., balls, juggling equipment, ropes, etc.) Role of teachers: Encouragement and supervision during recess and supported physical education classes to participate on the playground together with their students Control group: Business as usual	10 months	Counts per minute measured with accelerometers and SOPLAY (energy expenditure, proportion of children in MVPA) at baseline and 10 months after baseline
Kelly et al. [47], 2012, Australia	Non-RCT; primary school environment 4 schools (2 to the intervention group) 63/126 (mean age 6.5 years; 55% boys)	Intervention group: Coloured playground markings of the playground, equipment (bean bags, stop watch, balls), and games resource provided to teachers Role of teachers: Teachers taught games from the resource that children could play in the playground Control group: Business as usual	6 weeks	Time in SED, LPA, MPA, VPA, MVPA measured with accelerometers, observation of active and non-active children measured with the Children Activity Scanning Tool during break times and school day at baseline and 6 weeks after baseline
López-Fernándezet al. [48], 2016, Spain	Cluster-RCT; primary school environment 3 schools (1 to the intervention group) 68/148 (mean age 7.1 years; 63% boys)	Intervention group: Playground markings (four hopscotch), unfixed equipment (i.e., silicone flying discs, giant ball, traffic cone, etc.), play space divided into several line markings, each with equipment Role of teachers: No active role Control group: Business as usual	3 weeks	Number of steps measured with pedometers during recess at baseline and 3 weeks after baseline
Loucaides et al. [49], 2009, Cyprus	Cluster-RCT; primary school environment 3 schools (1 to the intervention group) 89/159 (mean age 11.1 years; 51% boys)	Intervention group: Provision of play space for team games, playground markings and long ropes for group jump rope and short ropes for solo jump rope Role of teachers: Instructed not to encourage children to participate in the activities Control group: Business as usual	4 weeks	Number of steps measured with pedometers during recess at baseline and 4 weeks after baseline
Ng et al. [50], 2020, Australia	Non-RCT; Preschool environment 11 pre-schools (6 to the intervention group) 159/297 (2–5 years old; 49% boys)	Intervention group: Major upgrade of their outdoor physical environment Role of teachers: Not defined Control group: Business as usual	12 months	Time in MVPA, TPA measured with accelerometers across the school day at baseline and 6 months after baseline
Nigg et al. [51], 2019, US	Cluster-RCT; primary school environment 24 schools (6 to the intervention group) 3009/6500 (overall mean age and gender% are not available)	Intervention group: Renovation of schoolyards, introducing community gateways and gathering spaces, public art works, age-appropriate play equipment, grass playing fields, colorful structured and unstructured asphalt games, custom shade structures, habitat areas, and nature play. Role of teachers: Not defined Control group: Business as usual	2 years	PA measured with SOPLAY (sedentary, walking or very active), self-report questionnaire, and accelerometers at baseline, and 1, 2, and 3 years after baseline
Ridgers et al. [6], 2010, UK	Non-RCT; primary school environment 26 schools (15 to the intervention group) 256/470 (mean age 8.1 years, 51% boys)	Intervention group: Three specific color-coded areas: a red sports area, a blue multi-activity area and a yellow quiet play zone; physical structures including soccer goal posts, basketball hoops and fencing; equipment, such as soccer balls, jump rope and tennis balls Role of teachers: Supervision Control group: Business as usual	12 months	Time in VPA, MVPA measured with accelerometers and heart rate monitor during recess at baseline, and 6 weeks, 6 months and 12 months after baseline
Stratton [7], 2000, UK Stratton & Leonard [39], 2002, UK	Cluster-RCT; primary school environment 2 schools (1 to the intervention group) 36/60 (5–7 years old; 50% boys)	Intervention group: Playground markings, linking to school curricular themes: castle, dragon, pirate ship, clock face, flower maze, fun trail and dens, hopscotch, letter squares, snakes and ladders, and a circular maze, evenly spaced throughout the playground area Role of teachers: Not defined Control group: No playground markings, but allowed limited equipment into the playground	4 weeks	Time in VPA, MVPA measured with heart rate telemetry during playtime at baseline and 4 weeks after baseline Energy expenditure measured with heart rate telemetry during play at baseline and 4 weeks after baseline It was not specified which outcome was the primary outcome of the experiment
Stratton & Mullan [52], 2005, UK	Non-RCT; primary school environment 8 schools (4 to the intervention group) 67/99 (7–11 years old; 52% boys)	Intervention group: Playground markings, linking to school curricular themes: castle, dragon, pirate ship, clock face, flower maze, fun trail and dens, hopscotch, letter squares, snakes and ladders, and a circular maze, evenly spaced throughout the playground area Role of teachers: Promoting physically active behaviour Control group: Business as usual	4 weeks	Time in MVPA, VPA measured with heart rate monitor during breaks at baseline and 4 weeks after baseline
Tucker et al. [53], 2017, Canada	Cluster-RCT; Preschool environment 22 childcare centres (11 to intervention group) 200/338 (2.5–4.5 years old; 52% boys)	Intervention group: Introduction of portable play equipment, e.g., balls, hula hoops, ribbon wands; re-structure of the two 60-min outdoor sessions into four 30-min periods Role of teachers Not defined Control condition: Business as usual	8 weeks	Time in SED, LPA, MVPA, TPA measured with accelerometers during childcare time at baseline, and 8 weeks, 6 months and 12 months after baseline
Verstraete et al. [54], 2006, Belgium	Cluster-RCT; primary school environment 7 schools (4 to the intervention group) 112/235 (fifth and sixth grade, 51% boys)	Intervention group: Provision of game equipment (e.g., ropes, flying discs, and balls) and ‘activity cards’ including examples of games and activities that can be performed with the equipment Role of teachers: Stimulate the children to play with the game equipment, divide the game equipment into different sets, and exchange those sets regularly to keep children interested Control group: Business as usual	3 months	Time in LPA, MPA, VPA, MVPA measured with accelerometers during recess at baseline and 3 months after baseline
**Other Outcomes: Movement behaviour, cognitive, psychological and social development**				
Crust et al. [55], 2014, UK	Non-RCT; primary school environment 16 schools (12 to the intervention group) 160/218 (10–11 years old; 54% girls)	Intervention group: Marking of the playground with colored lines, shapes, and boxes. Role of teachers: Promote and support children in the use of the markings Control school: Business as usual	8 months	Primary outcome: physical self-concept and self-esteem measured with the Children and Youth Physical Self-Perception Profile at baseline and 8 months after baseline Secondary outcome: Social interactions measured with SOCARP
Kelz et al. [56], 2015, Austria	Non-RCT; secondary school environment 3 schools (1 to the intervention group) 72/195 (13–15 years old, 51% boys)	Intervention group: Implementation of green areas (a hedge consisting of 10 shrubs and 10 pot plants around the seating area), seating (9 wooden tables and chairs, 3 wooden benches as well as 15 seating pillows), sports opportunities (2 soccer goals and field markings, 2 table tennis tables, 1 volleyball net), and a drinking fountain Role of teachers: Not defined Control group: Business as usual	3 months	Executive function measured with the Attention Network Test Well-being measured with The Basler Well-Being Questionnaire It was not specified which outcome was the primary, and the outcomes are here ordered as in the study
Raney, et al. [57], 2021, US	Non-RCT; primary school environment 2 schools (1 to the intervention group) 642/1031(first to fifth grades, gender % not reported)	Intervention group: Playground line markings were divided in spaces, and were replaced by green space, including (1) introduction of trees, mulch, and boulders in two line markings; (2) replacement of one asphalt field with grass and trees; (3) replacement of another asphalt field with an outdoor classroom (decomposed granite floor, mulch and plant border, and log seating) Role of teachers: Not defined Control group: Business as usual	16 months	Time in SED, LPA, MVPA measured with accelerometers and SOPLAY at baseline, after renovation and 4 months and 16 months after baseline Social interactions were measured with SOCARP It was not specified which outcome was the primary, and the outcomes are here ordered as in the study
Sanz-Mas et al. [58], 2025, Spain	Non-RCT; secondary school environment 21 schools (11 to the intervention group) 407/792 (11–12 years old; 45%)	Intervention group: Redesign of schoolyards: planting trees, adding green walls with climbing plants, and creating gardens with Mediterranean species while replacing hard surfaces in schoolyards with more natural elements, inclusion of fountains for drinking, playing, and cooling purposes, and installing pergolas, canopies, and seating areas Role of teachers: Not defined Control group: Business as usual	9 months	Social behaviour measured with a custom-made questionnaire at baseline and 9 months after baseline
Van Dijk-Wesselius et al. [59], 2018, The Netherlands	Non-RCT; primary school environment 9 schools (5 to the intervention group) 2031 (7–11 years old; 49% boys)	Intervention group: Greening of schoolyards (e.g., grassy hills, bushes, tree, tunnels made of tree branches, loose tree branches and garden-like parts) Role of teachers: Not defined Control group: Business as usual	2 years	Time in MVPA measured with accelerometers at baseline, and 1 year and 2 years after baseline Attention measured with DLST and SST Prosocial orientation with The Social Orientation Choice Card Self-reported social behaviour with Subscales Peer problems and Prosocial behaviour, and subscale social support in friendships Self-perceived emotional functioning with the Paediatric Quality of life scale It was not specified which outcome was the primary, and the outcomes are here ordered as in the study

Abbreviations: *SED* sedentary behaviour, *LPA* light physical activity, *MPA* moderate physical activity, *VPA* vigorous physical activity, *MVPA* moderate to vigorous physical activity, *TPA* total physical activity, *PA* physical activity, *SOCARP* System for Observing Children’s Activity and Relationships during Play,* PRAQ-R* Peer Relations Assessment Questionnaires–Revised*, SOPLAY *System of Observing Play and Leisure Activities in Youth, *Peds QL 4.0* Pediatric Quality of Life Inventory,* DLST* Digit Letter Substitution Test, *SST* Sky Search task

Seventeen studies adopted a cluster-RCT design [7, 8, 35–42, 45, 46, 48, 49, 51, 53, 54], while 11 studies adopted a quasi-experimental design (i.e., without randomization) [6, 43, 44, 47, 50, 52, 55–57, 59, 60]. All studies employed a baseline and post-intervention assessment design. The interventions and the assessment time-points spanned durations from 2 weeks to 2 years. This heterogeneity was analysed and partitioned using meta-regression and subgroup analyses. Ten studies conducted multiple post-intervention measurements [6, 35, 36, 40, 44, 50, 51, 53, 57, 59], and it was not specified which time point was the primary point of interest. Three of these 10 studies [6, 45, 57] reported outcomes at the different time points in different articles [31–34]. In all studies, experimental groups were compared to control groups which did not make any change to their playground environment and continued business as usual. Some studies included multiple experimental and/or control groups. For these studies, only the group that implemented physical environmental changes only and the control group that continued business as usual were included. One study [42] had 3 experimental groups with physical enrichment (marking, equipment, and a combination of the two), and the results were averaged across the 3 experimental groups for the meta-analysis.

The included studies assessed a variety of outcomes, including movement behaviours, cognitive, psychological and social development. Movement behaviour was the primary outcome for most of the studies (*n* = 23 [6–8, 35–54]), psychological development was the primary outcome in 1 study [55], social development was the primary outcome in 1 study [60], and the primary outcome was not specified in 3 studies [56, 57, 59].

#### Movement behaviours

Twenty-three studies evaluated movement behavioural outcomes (sedentary time and physical activity), which were measured with accelerometers (ActiGraph GT1M, GT3X, MTI, and Actical) [6, 8, 36, 38, 40–43, 45–47, 50, 53, 54, 57, 59], pedometers (Dista Newfeel 100, DW-200, and Yamax Digiwalker SW200) [44, 48, 49], heart rate telemetry (Polar Team, and Sportstester) [6, 7, 39, 52], or an observational tool (SOPLAY) [44–46, 51, 57]. The outcome measures included (i) sedentary time (SED; 10/28 studies), light physical activity (LPA; 9/28), moderate physical activity (MPA; 4/28), vigorous physical activity (VPA; 7/28), and moderate-to-vigorous physical activity (MVPA; 18/28), (ii) number of steps (3/28), and (iii) energy expenditure (1/28).

####  Cognitive development

Cognitive developmental outcomes were evaluated in 2 studies. Executive functioning was measured with the Attention Network Test [56], the Digit Letter Substitution Test and the Sky Search task [59], and the outcomes included a score of attentional functioning.

#### Psychological development

Psychological developmental outcomes were evaluated in 3 studies. Self-concept and self-esteem were measured with the Children and Youth Physical Self-Perception Profile [55]. Quality of life was measured with the Pediatric Quality of Life Inventory 4.0 [44]. Lastly, emotional functioning was measured with the Pediatric Quality of Life scale [59].

#### Social development

Social developmental outcomes were evaluated in 5 studies. Social interactions were measured using video recordings and live observation with the System for Observing Children’s Activity and Relationships during Play tool [37, 55, 57], and the outcome measures included the quantity and type of social interactions amongst individuals. The perception of social acceptance was measured with the Pictorial Scale of Perceived Competence and Social Acceptance for Young Children [37]. Social behaviour during play was measured with a custom-made questionnaire [60]. Bullying was measured with the Peer Relations Assessment Questionnaires–Revised [35].

All studies implemented complexity, with 6 studies implementing only objects, such as portable equipment (e.g., ball, ropes, bean bags, and car tyres) [37, 38, 44, 45, 53, 54]; 4 studies line markings only to divide the playground in activity and game areas [7, 39, 52, 55]; 7 studies objects and line markings [40–42, 46–49]; 7 studies objects, line markings, and structures (e.g., climbing frames and monkey bars) [8, 35, 36, 43, 50, 51, 56], 3 studies structures and line markings [6, 57, 59], and 1 study structures only [60]. All studies implemented variety, enriching the playground with a variety of possible activities, such as running, jumping, climbing, and skipping. Four studies implemented novelty [37, 44, 46, 54], and 12 studies involved end-users in their intervention design [7, 8, 35, 36, 39, 42, 43, 51–54, 59, 60]. Children, teachers, children’s parents, and school communities were considered as relevant end-users as they would directly or indirectly benefit from the intervention and can provide meaningful input to tailor the intervention to children’s needs and preferences. Only 1 study implemented scaffolding by progressively introducing the enriching equipment weekly to avoid overstimulation [44] (Table 2). Five studies added pedagogical strategies to encourage children to engage with the physical modification. The pedagogical strategies included the provision of game resources to teachers [47], linking playground markings to different child-friendly themes [7, 39, 52], and provision of activity cards that show examples of activities children could perform in the playground [54]. While studies with a large focus on teachers’ role in their intervention (e.g., direct teaching) were excluded, the teachers had different roles across the included studies: they had a supervisory role [6, 40, 44], introduced the equipment and made it available to children [42], taught children games they could play in the playground [47], encouraged children to be active [8, 46, 52], managed the material throughout the intervention [54], encouraged children to engage with the modified environment [55], or did not have a defined [7, 35–39, 41, 43, 50, 51, 53, 56, 57, 59] or active role [45, 48, 49].

**Table 2:** The 5 enrichment themes implemented in the included studies

	Complexity	Variety	Novelty	Needs targeted and end-users involved	Scaffolding
Baquet et al., 2018 [40]	Yes (objects and line markings)	Yes	No	Need: Play End-user: None	No
Blaes et al., 2013 [41]	Yes (objects and line markings)	Yes	No	Need: Play End-user: None	No
Bundy et al., 2017 [37]; Engelen et al., 2013 [38]	Yes (objects)	Yes	Yes	Need: Play and creativity End-user: None	No
Cardon et al., 2009 [42]	Yes (objects and line markings)	Yes	No	Need: Play End-user: Teachers	No
Christiansen et al., 2017 [8]	Yes (objects, structures, line markings)	Yes	No	Need: Play End-user: Children	No
Crust et al., 2014 [55]	Yes (line markings)	Yes	No	Need: Play End-user: None	No
Farmer et al., 2017 [35, 36]	Yes (objects, structures, line markings)	Yes	No	Need: Play and risk-taking End-user: School community	No
Hamer et al., 2017 [43]	Yes (objects, structures, line markings)	Yes	No	Need: Play End-user: Children and teachers	No
Huberty et al., 2014 [45]	Yes (objects)	Yes	No	Need: Play End-user: None	No
Hyndman et al., 2014 [44]	Yes (objects)	Yes	Yes	Need: Play and creativity End-user: None	Yes
Janssen et al., 2015 [46]	Yes (objects and line markings)	Yes	Yes	Need: Play End-user: None	No
Kelly et al., 2012 [47]	Yes (objects and line markings)	Yes	No	Need: Play End-user: None	No
Kelz et al., 2013 [56]	Yes (objects, structures, line markings)	Yes	No	Need: Play End-user: None	No
López-Fernández et al., 2016 [48]	Yes (objects and line markings)	Yes	No	Need: Play End-user: None	No
Loucaides et al., 2009 [49]	Yes (objects and line markings)	Yes	No	Need: Play End-user: None	No
Ng et al., 2020 [50]	Yes (objects, structures, line markings)	Yes	No	Need: Play End-user: None	No
Nigg et al., 2019 [51]	Yes (objects, structures, line markings)	Yes	No	Need: Play End-user: Children, teachers and parents	No
Raney et al., 2021 [57]	Yes (structures, line markings)	Yes	No	Need: Play End-user: None	No
Ridgers et al., 2010 [6]	Yes (structures, line markings)	Yes	No	Need: Play End-user: None	No
Sanz-Mas et al., 2025 [60]	Yes (structures)	Yes	No	Need: Play and shading End-user: School community	No
Stratton, 2000 [7]; Stratton & Leonard, 2002 [39]	Yes (line markings)	Yes	No	Need: Play End-user: Children	No
Stratton & Mullan, 2005 [52]	Yes (line markings)	Yes	No	Need: Play End-user: Children	No
Tucker et al., 2017 [53]	Yes (objects)	Yes	No	Need: Play End-user: Children	No
van Dijk-Wesselius et al., 2018 [59]	Yes (structures, line markings)	Yes	No	Need: Play End-user: Children, teachers and parents	No
Verstraete et al., 2006 [54]	Yes (objects)	Yes	Yes	Need: Play End-user: Children	No

### Risk of Bias in the included studies

**Fig. 2:**
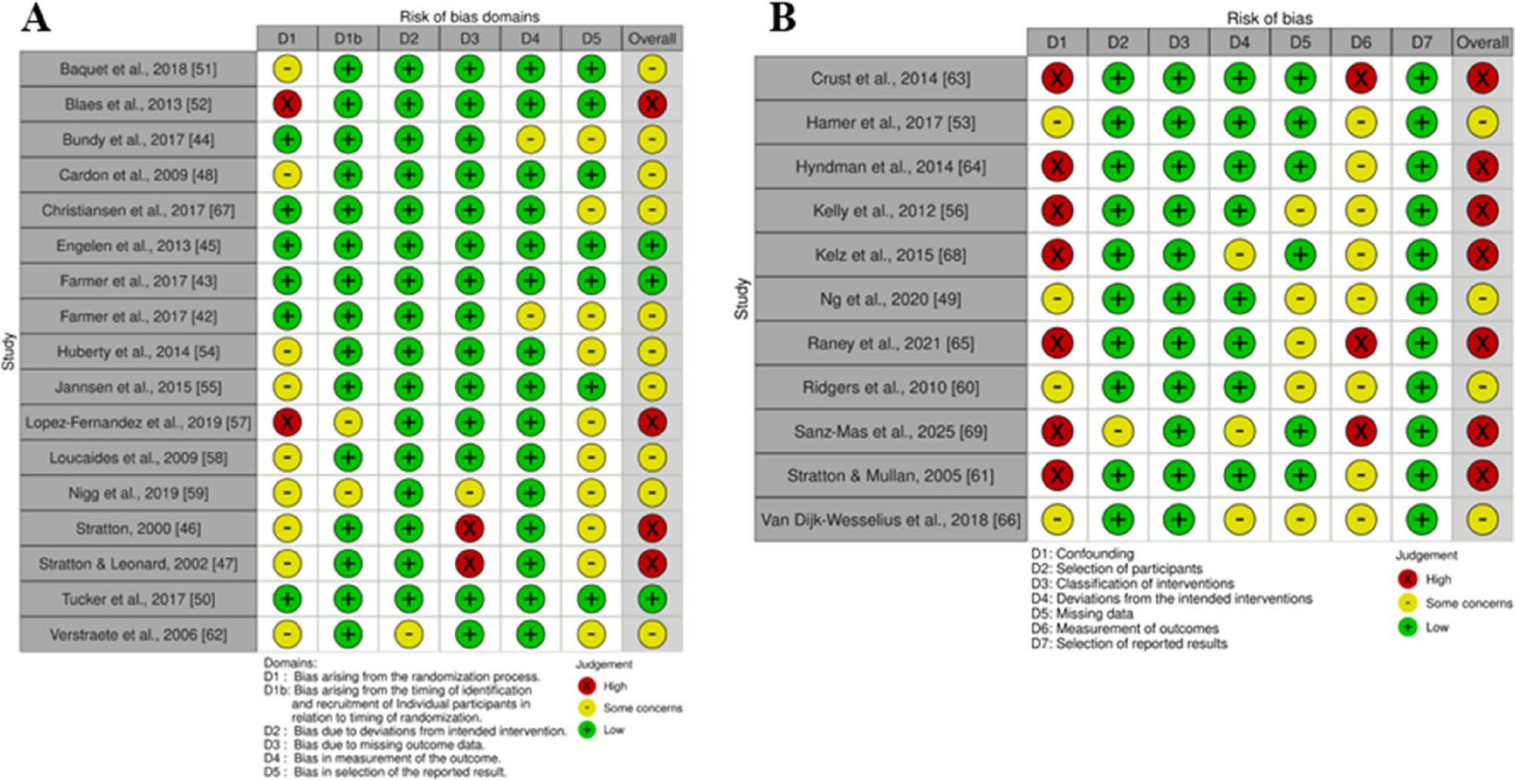
Risk of Bias (RoB) of the included studies using the traffic light visualization. Panel A shows the RoB of the cluster-RCT studies, and panel B shows the RoB of the quasi-experimental studies

The risk of bias of the included studies is presented in Fig. 2 using the traffic light system, with the green, yellow, and red colours representing low risk, some concerns, and high risk respectively. Across the cluster-RCTs, 3 studies were considered to have low risk [36, 38, 53], 10 studies to have some concerns [8, 35, 37, 40, 42, 45, 46, 49, 51, 54], and 4 studies to have high risk of bias [7, 39, 41, 48] (Fig. 2, panel A). The randomization procedure represented the domain with the highest concerns or high risk of bias. Across the quasi-experimental studies, 4 studies presented overall some concerns [6, 43, 50, 59] and 7 a high risk of bias [47, 52, 55–57, 60] (Fig. 2, panel B). The confounding and measurement of outcome domains were the most problematic, as most studies did not define and control for potential confounding variables and did not include blinding of subjective measurements.

###  Synthesis of results

A meta-analytic synthesis of results was only possible for the movement behaviour outcomes as there was few studies for other outcomes. A narrative synthesis is presented for the other outcomes.

#### Movement behaviours

**Fig. 3:**
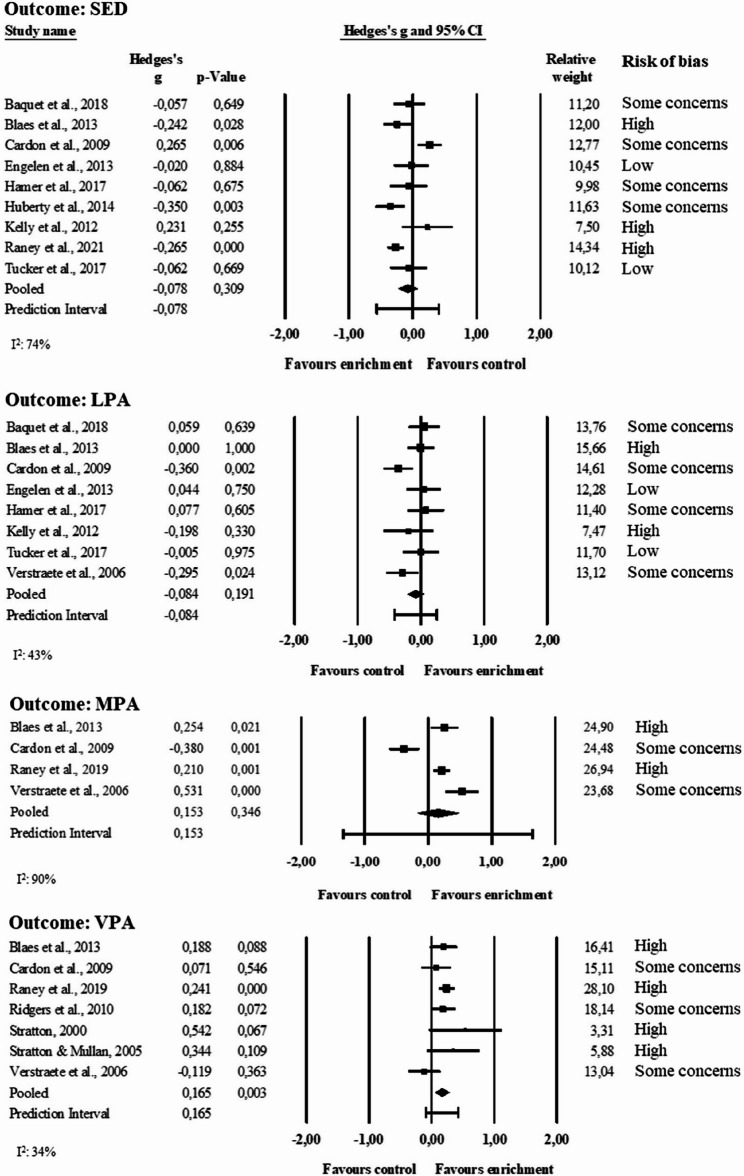
Forest plots of the meta-analyses of SED, LPA, MPA, and VPA. Results are reported as standardized mean differences (Hedge’s g)

The forest plots in Fig. 3 show the pooled effect sizes for SED (n = 9 studies included [38, 40–43, 45, 47, 53, 57], 3356 participants), LPA (n= 9 studies [38, 40–43, 47, 53, 54], 1971 participants), MPA (n = 4 studies [41, 42, 54, 57], 1889 participants), and VPA (n = 7 studies [6, 7, 41, 42, 52, 54, 57], 2429). The effect was statistically significant only for VPA (g = 0.17, 95% CI 0.06 to 0.27, p < 0.01) and it was a very small effect. The prediction interval of VPA indicates that the true effect is likely to be positioned within−0.09 and 0.42 SDs, spanning trivial to moderate effect in favour of enrichment. It must be noted in the VPA analysis that 4 studies out of 7 had a high risk of bias. The prediction intervals and I^2^ statistics indicate low-to-moderate heterogeneity for VPA (I^2^= 34%), as well as LPA (I^2^= 43%), and substantial heterogeneity for SED (I^2^= 74%) and MPA (I^2^= 90%) (thresholds for I^2^ according to Cochrane guidelines [61]). The other measures of heterogeneity are provided in the Supplementary Materials.

Inspecting the plots of SED, it can be observed that line markings (after 2 weeks [41] and 16 months [57]) and provision of equipment (after 8–9 months [45]) drive the effect in favour of enrichment in primary schools, that the provision of equipment combined with line markings drive the effect in favour of control in pre-schools (after 4–6 weeks [42]), and that the others did not show any effect. For LPA, the provision of equipment combined with line markings in pre-schools (after 4–6 weeks [42]) and the provision of equipment in primary schools (after 3 months [54]) drive the effect in favour of control, while the other strategies have no effect. For MPA, line markings (after 2 weeks [41] and 16 months [57]), and provision of equipment (after 3 months [54]) drive the effect in favour of enrichment in primary schools, while the provision of equipment combined with line markings (after 4–6 weeks [42]) drive the effect in favour of control in pre-schools. For VPA, line markings combined with equipment (after 2 weeks, [41]) or structures (after 12 months [6] and 16 months [57]), and line markings alone (after 4 weeks [7]) drive the effect in favour of enrichment in primary schools.

**Fig. 4:**
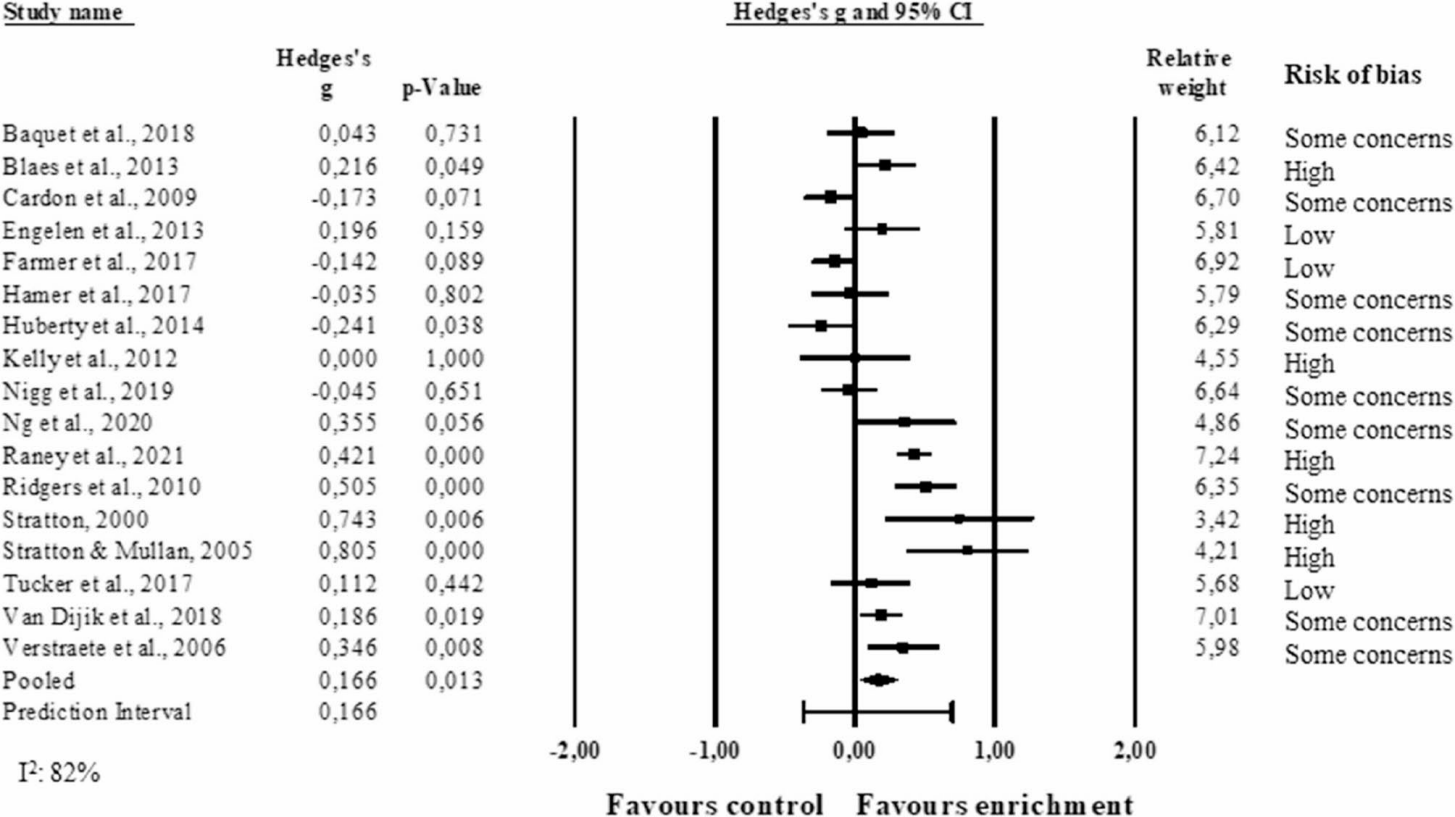
Forest plot of the meta-analysis of moderate-to-vigorous physical activity. Results are reported as standardized mean differences (Hedge’s g)

The forest plot in Fig. 4 shows the pooled effect size for MVPA (n = 17 studies [6, 7, 36, 38, 40–43, 45, 47, 50–54, 57, 59], 6859 participants), which was statistically significant (g = 0.17, 95% CI 0.03 to 0.30, p= 0.01) and represented a very small effect. Two additional studies were sought to be included through email contact with the authors; however, the data were not available [8, 46]. The prediction interval indicates that the true effect is likely within −0.37 and 0.70 standard deviations, spanning moderate effect in favour of control and large effect in favour of enrichment. The I^2^ statistics was equal to 82% confirming a substantial heterogeneity. Five studies out of 17 had a high risk of bias.

A moderation analysis was conducted on MVPA (not enough sample for the other outcomes) to better understand the results and inspect the source of the large heterogeneity. Potential sources of heterogeneity and moderators in the analysis are complexity type, involvement of end-users, total number of design themes considered, assessment time point, and school setting (pre-school, primary and secondary school). These, except for time point (there was an issue with collinearity) were used as covariates in the meta-regression. The meta-regression model (Q = 43.8, df = 7, p < 0.01) showed a statistically significant effect for complexity type (p < 0.01), involvement of end-users (p< 0.01), and the number design themes considered (p < 0.01), while school setting did not have a statistically significant effect (p = 0.18). This model explained 88% of the variance (R^2^) (Table 3). The coefficients indicate that line markings, and structures and line markings from the complexity type covariate increased the effect size, the involvement of end-users decreased the effect size, and the number of themes increased the effect size (the higher the number of themes the higher the effect size). An incremental model – adding each covariate individually and incrementally – showed that the total R^2^ was partitioned into R^2^ of 0.59 from complexity type, R^2^ of 0.07 from the involvement of end-users, and R^2^ of 0.21 from the number of themes considered, and R^2^ of 0.02 from school setting.

**Table 3:** Results of the meta-regression with complexity type, involvement of end-users, number of design themes considered, and school setting implemented as covariates

Covariate	Coefficient	Standard Error	95% Lower	95% Upper	Z-value	*P*-value
Intercept	−0.99	0.42	−1.81	−0.16	−2.33	0.02
Complexity type: objects	0.16	0.13	−0.10	0.42	1.20	0.23
Complexity type: objects, line markings	0.02	0.12	−0.21	0.25	0.20	0.84
Complexity type: structures, line markings	0.42	0.12	0.19	0.66	3.56	< 0.01
Complexity type: line markings	1.02	0.22	0.59	1.45	4.64	< 0.01
Involvement of end-users	−0.86	0.25	−1.35	−0.36	−3.41	< 0.01
Number of themes	0.59	0.20	0.20	0.98	2.95	< 0.01
School setting	−0.16	0.12	−0.39	0.07	−1.36	0.17
Test of the model: Q = 43.81, df = 7, *p* < 0.01						
Proportion of variance explained by the model: R^2^ = 0.88						

**Fig. 5:**
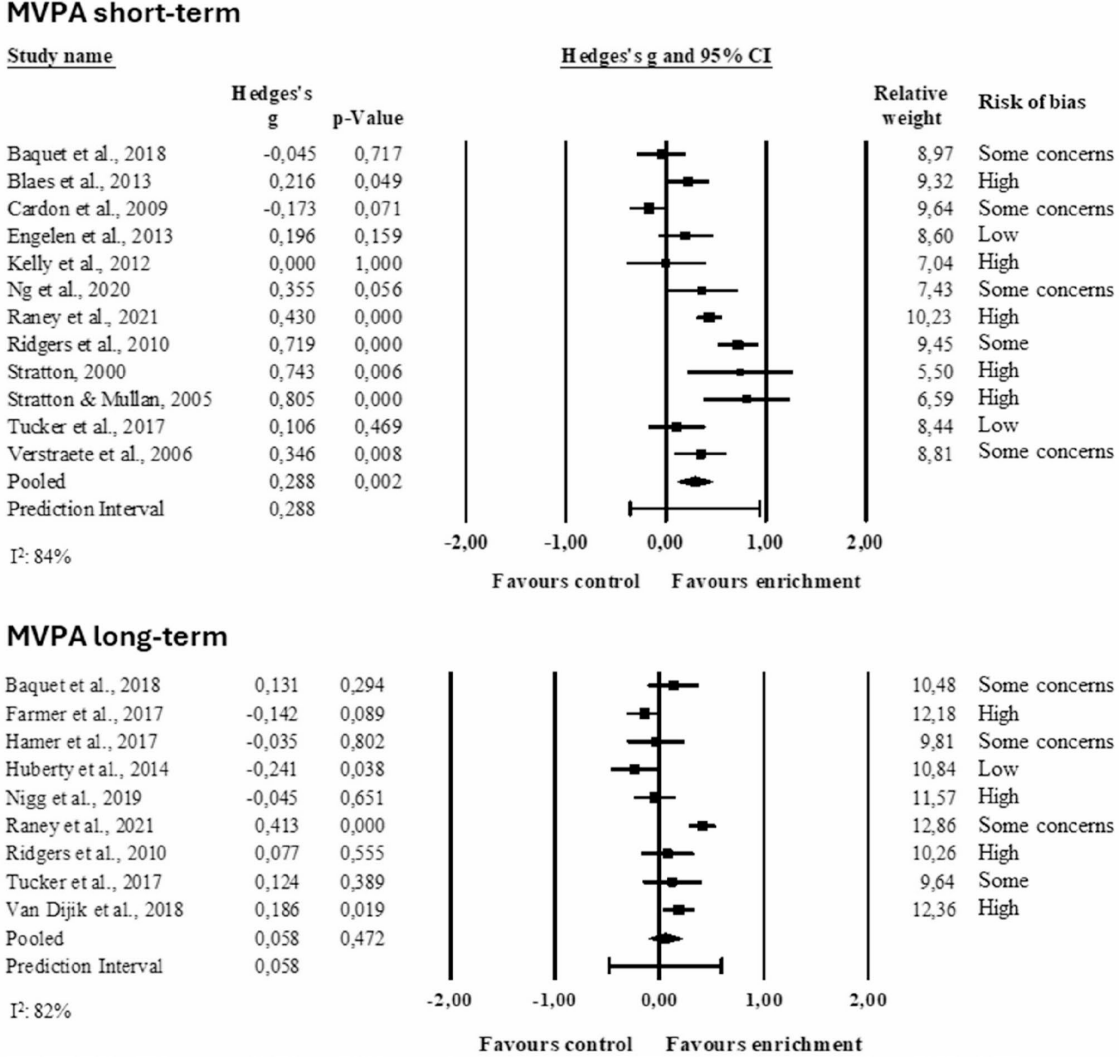
Forest plot of sub-group analysis based on assessment time point on moderate-to-vigorous physical activity. Results are reported as standardized mean differences (Hedge’s g)

A sub-group analysis was performed to evaluate the effect of assessment time point. With a cut-off of 12 months, it was created a short-term group (studies with a time point below 12 months) and a long-term group (studies with a time point equal to or above 12 months). The meta-analysis showed a statistically significant effect in the short-term group only (g = 0.29, 95% CI 0.11 to 0.47, p < 0.01), with a prediction interval ranging from −0.37 to 0.94 (Fig. 5). This indicates an effect of time point.

The sensitivity analysis was performed on MVPA and VPA, showing no effect of any individual study on the 2 outcomes (see the Supplementary Materials). 

 The publication bias analysis did not show any bias for SED, LPA, MPA, VPA, or MVPA, as the Trim and Fill method did not detect any additional study, and the funnel plots did show any asymmetry around the pooled effect size (see the Supplementary Materials).

#### Other movement behaviour outcomes

**Fig. 6:**
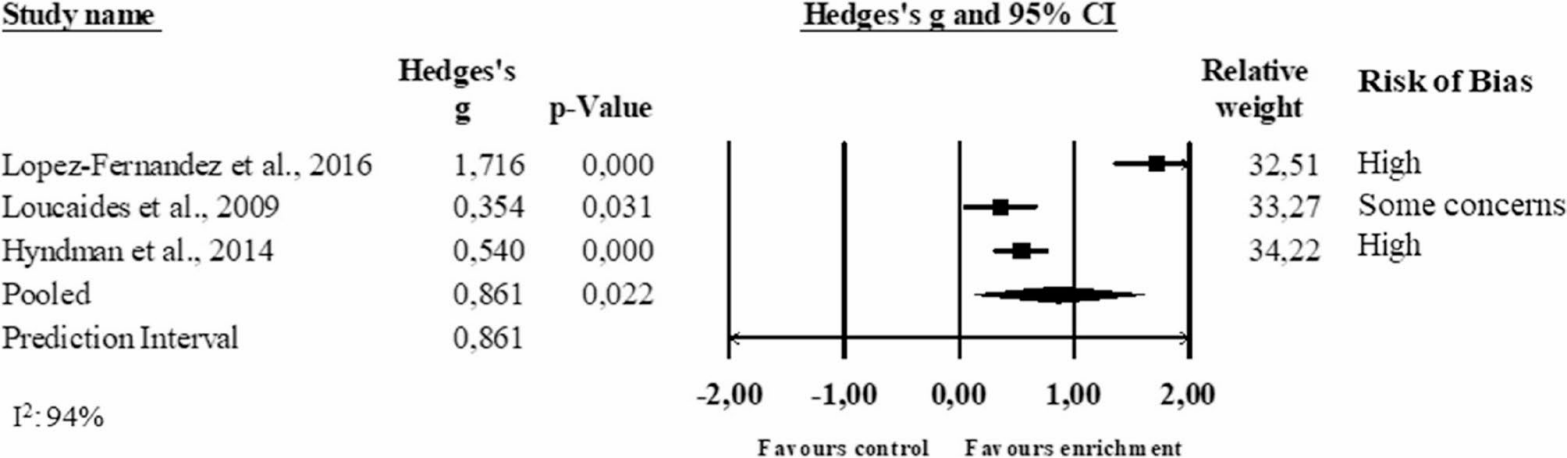
Forest plot of the meta-analysis of the number of steps per minute. Results are reported as standardized mean differences (Hedge’s g)

Three studies were included in the analysis of the number of steps per minute, with a total of 573 participants. The computed effect size (Hedges’ g) was 0.86 (95% CI 0.12 to 1.60, p = 0.04), and the prediction interval ranged from −8.47 to 10.19 (Fig. 6). The provision of equipment in isolation (8 months assessment point, [44]), and in combination with line markings (3 weeks assessment point, [48], 4 weeks assessment point, [49]) increased the number of steps in primary schools.

##### Level of evidence

The level of evidence of the movement behaviour outcomes was low, due to limitations related to the risk of bias and imprecision of the results (see the Supplementary Materials).

#### Cognitive development

van Dijk-Wesselius et al. [59] detected a significant effect of enrichment on attention restoration in the Digital Letter Substitution Test and a non-significant improvement (p = 0.08) in the intervention group in the Sky Search task, while Kelz et al. [56] did not find any effect of enrichment on executive functioning.

#### Psychological development

Kelz et al. [56] found an intervention effect on intra-psychic balance and overall wellbeing, while Hyndman et al. [44] did not find an intervention effect on psychosocial quality of life and overall quality of life. 

#### Social development

Raney et al. [57] found an effect of enrichment on increasing prosocial interactions and decreasing antisocial interactions. Similarly, Crust et al. [55] reported (but did not provide statistical details of) a 6.7% increase in prosocial interactions. van Dijk-Wesselius et al. [59] did not find an overall effect of enrichment on prosocial orientation, and found that self-reported social support increased only in grade 5 and 6. Contrarily, Bundy et al. [37] found no effect of enrichment on perceived social acceptance or social skills, and similarly Sanz-Mas et al. [60] did not find any significant effect of enrichment on social behaviour (i.e., playing alone and in groups). Lastly, Farmer et al. [35] did not find any effect of enrichment on bullying.

## Discussion

This study reviewed the effects of physical enrichment of (pre-)school playgrounds on children’s and adolescents’ movement behaviours, and their cognitive, psychological, and social development. The included studies presented high heterogeneity of environmental exposures and design of the experimental investigation and were overall of low methodological quality. This must be considered when interpreting the findings. The included studies focused almost exclusively on physical activity level and meta-analysis was possible only for these outcomes. The meta-analyses showed small, statistically significant increases in VPA, MVPA, and number of steps in the enrichment interventions relative to the control conditions, but no statistically significant effects for SED, LPA, and MPA. The results for MVPA had high heterogeneity. While publication bias was not present for any of these outcomes, sensitivity analyses did not show any effect of removing any individual studies, it must be noted that some of the studies for VPA, MVPA, and steps had a high risk of bias; 4/7, 5/17, and 2/3 of the studies, respectively. All the outcomes considered had a low level of evidence. The evidence for social, cognitive, and psychological development is inconclusive, as the narrative synthesis showed conflicting results for these outcomes. For instance, some studies suggested a potential role of enrichment in facilitating social interactions among children and youth, while other studies did not find any effects for development of social skills or perceived social skills. The meta-regression and sub-group analysis performed for MVPA and the narrative synthesis conducted for VPA partitioned their high heterogeneity across studies and analysed the effects of the different enrichment strategies. These analyses showed that enrichment strategies differentially influenced the effect. Line markings alone or in combination with physical structures increased MVPA, while equipment and line marking plus equipment did not have an effect. Line markings alone or in combination with equipment or physical structures increased VPA. The sub-group analysis for MVPA showed that the effect was larger at short term than at long term. These results slightly differ from previous reviews on school playground design. While Broekhuizen et al. [9] found moderate evidence of the provision of play equipment and inconclusive or no evidence of line markings and play line markings, Escalante et al. [12] found that line markings in combination with physical structures increase PA level and equipment and line markings in isolation do not. The current review in contrast showed small effect of line markings and line markings in combination with physical structures or equipment on MVPA and VPA. The different results could be due to the current review including more studies and synthesizing the results objectively through a meta-analysis complemented with a meta-regression. The meta-regression on findings for MVPA showed how the involvement of end-users influenced the effect. Contrary to what one might hypothesize, the involvement of end-users decreased the effect. However, studies did not provide detailed information on how the end-users were involved, what insights they provided, and how their insights shaped the intervention design, making it difficult to fully interpret these findings. Considering that most studies involved children in the design process, we speculate that children may provide their preferences for themes and colours of the playground modifications, but not so much about modifications to achieve the intended developmental outcomes. Consistent with previous literature [62, 63], teachers and school communities may more broadly provide suggestions on modifications tailored to their children aiming to promote developmental outcomes. Perhaps the involvement of multiple end-users, including children, teachers, and school communities could prove beneficial in future studies to increase the effect of enrichment interventions.

### Practical implications 

School playgrounds represent a key environment and opportunity to enhance children’s health and their overall development [64], and the current review provides some evidence in support of enriching them. While changes to the physical aspects of a playground may raise practical concerns over the cost of such interventions, the current findings show how relatively simple modifications can increase physical activity levels in children and youth. Some interventions were expensive, especially the ones that renovated the playground (e.g., up to 300k Euro per school in [8]), but others were more manageable (e.g., between 10k and 20k Euro per school to provide markings and equipment [6, 35, 36, 40, 41]), and some were almost cost-free (e.g., use of recycled material [37, 38, 44] and markings painted by the researchers or school staff [42, 52]) (see the Supplementary Materials for the list of intervention cost). Interestingly, line markings and the creation of play zones, which can be very cheap, were the most effective strategies. Taken together, this highlights the potential sustainability of playground modifications, which, based on the results of this review, are mostly pedagogical and not structural. In summary, these findings can inform policy makers highlighting the importance of careful design of the physical environments and that they should not invest in a one size fits all approach to playground design.

### Future directions – how the environmental enrichment paradigm can advance current practice 

Currently, a clear framework for designing physical enrichment of school playgrounds is lacking. Grounded in the Environmental Enrichment (EE) paradigm, a recent review provided a pedagogical framework for evaluating and informing the design of environmental changes that can be adopted in human contexts [17]. EE is a key experimental paradigm in biology and neuroscience, widely used in animal-based research. It is typically achieved by enlarging the animals’ space, adding objects (e.g., toys) and equipment (e.g., running wheel) to increase opportunities for movement, exploration, and sensorimotor stimulation as well as adding additional rodents to promote socialization [65, 66]. Enriching a school’s physical environment therefore can be considered a form of EE similar to that seen in the animal studies. 

 Oppici et al. [17] review identified a series of EE principles that can inform the design of environmental changes with the potential of applying them to physical enrichment of school playground: complexity, variety, novelty, targeting needs, and scaffolding. As has been shown in this review these principles can be used to provide researchers a framework to better understand the process of enriching playful spaces. The enrichment process entails building complexity and variety of behavioural opportunities, keeping up the stimulation through novelty, involving end-users to tailor intervention to their needs and context, and scaffolding the modifications to avoid overstimulation and ensure adherence. While all the studies included in the review implemented complexity and variety, and half of them involved end-users, just a handful of studies (n = 4) implemented novelty and only 1 study implemented scaffolding. 

Novelty and scaffolding are expected to have a significant impact and increase the effect of environmental modification on movement behaviours [17]. The meta-regression showed that implementing a higher number of these principles increased MVPA, suggesting that EE principles may indeed improve the design of physical enrichment of school playgrounds. Novelty is a key ingredient for the EE paradigm [67]. It contributes to maintaining a high level of engagement of the animals with the presented stimuli and promotes increased levels of physical activity [68]. This could well apply to playground design. For instance, changing regularly (e.g., weekly) the game and activity line markings of the playground will encourage children to move around and explore the new opportunities those changes offer. This can be especially important considering the results showing a decrease in effect in the long term. Novelty could be the key to maintain the enhanced level of MVPA observed in the short term. 

 While scaffolding is a new term in the EE literature [17], it is well established in teaching and learning education [69]. Scaffolding consists of providing support tailored to the students’ abilities that fades away and gradually gives independence to the learner. This could be applied in playground interventions by tailoring interventions to students’ abilities and preferences, gradually introducing the enriching elements, supporting students initiating their interaction with EE elements and then giving them independence to modify the playground. This scaffolding strategy is likely to increase students’ motivation and engagement with intervention, ultimately enhancing their activity.

Another lesson we can learn from the EE paradigm is the combined assessment of physical activity level and movement skills (e.g., proficiency in catching and throwing). The EE literature indicates that modifications of the environment providing a range of movement opportunities have substantial effects on movement skills [58, 70]. For example, the studies included in this review did not assess movement skills and might have missed the impact of adding objects to the playground. Whilst children interact with both fixed and moveable objects, MVPA was not affected but movement skills are likely to have improved. This further reinforces the benefits of incorporating the whole EE paradigm to improve the design of playground interventions and the measurement of outcomes. The systematic implementation of the EE-grounded principles may also contribute to reducing heterogeneity in future research.

### Limitations of this review

This review presents some limitations that are worth considering. While the effect sizes are pooled from a relatively large number of studies and participants, the large heterogeneity in the effect size and the low level of evidence warrant some caution in the interpretation of the results. This highlights the need for additional well-designed experimental research. Future research should focus on more transparent and systematic recruitment and randomization procedures, as most of the included studies presented issues in these areas. Further, most of the cluster-RCTs included had a low number of clusters, and power calculation was not performed in any of the studies. These are certainly aspects that future research should improve. More evidence-based approach to the design of intervention duration and assessment time point would improve future research, as the included studies had large variations on these aspects contributing to the substantial heterogeneity of the meta-analysis results. Considering the principle of novelty could initially guide researchers’ decision in their design of short- or long-term interventions.

## Conclusion

This review provides weak evidence for the effect of physical enrichment of (pre-)school playground on increasing VPA, MVPA, and number of steps in pre- and primary schools. Specifically, line markings alone or in combination with physical structures increased MVPA, while line markings alone or in combination with physical structures or equipment increased VPA. Interestingly, involving end-users decreased MVPA. The enrichment effect on MVPA was significant only in the short-term. Due to few studies and conflicting results, the evidence for social, cognitive, and psychological development were inconclusive. The studies included in this review had low methodological quality (only 3/28 studies presented low risk of bias) and their type of enrichment and findings were largely heterogeneous. Thus, while this review provides initial evidence for schools, practitioners, and policymakers on the use of physical enrichment of school playgrounds for tackling physical inactivity, more studies are needed to inform research and practice.

## Supplementary Information

Supplementary Material 1.

Supplementary Material 2.

Supplementary Material 3.

Supplementary Material 4.

Supplementary Material 5.

Supplementary Material 6.

Supplementary Material 7.

Supplementary Material 8.

Supplementary Material 9.

## Data Availability

The dataset for the meta-analysis is provided in the Supplementary Materials.

## Abbreviations

EE: Environmental enrichment
PRISMA: Preferred reporting items for systematic reviews and meta-analyses
PICOS: Population, intervention, comparator, outcome, study design
RCT: Randomized controlled trial
CI: Confidence interval
SMD: Standardized mean difference
SD: Standard deviation
GRADE: Grading of recommendations assessment, development and evaluation
SED: Sedentary behaviour
LPA: Light physical activity
MPA: Moderate physical activity
VPA: Vigorous physical activity
MVPA: Moderate to vigorous physical activity
TPA: Total physical activity
SOCARP: System for observing children’s activity and relationships during play
PRAQ-R: Peer relations assessment questionnaires–revised
SOPLAY: System of observing play and leisure activities in youth
Peds QL 4.0: Pediatric quality of life inventory
DLST: Digit letter substitution test
SST: Sky search task

## Footnotes

1 These outcomes were slightly modified from the protocol registered in PROSPERO to better capture the behaviours expected to be affected from physical enrichment.
