# Supplementary material for: How can physical enrichment of school playgrounds improve movement behaviours and developmental outcomes in children and adolescents? A systematic review with meta-analysis
Source: Int J Behav Nutr Phys Act. 2025 Nov 22;22:161. doi: 10.1186/s12966-025-01856-y (PMC12751770; doi:10.1186/s12966-025-01856-y)
Supplement: Supplementary file 2 — Supplementary Material 2. [file 12966_2025_1856_MOESM2_ESM.docx]

Inclusion and exclusion criteria

| **Criteria** | **Inclusion** | **Exclusion** |
| --- | --- | --- |
| Population | 2- to 18-year-old typically developing individuals attending education in early years and/or school environments | Participants beyond the age range or from specific populations |
| Intervention | physical enrichment of playful environments in the school context, such as line markings and the addition of equipment, as the main intervention strategy | Interventions conducted in settings beyond school, and multi-component interventions with a strong role of the teacher, such as enrichment combined with a new pedagogical approach to increase students’ activity. |
| Comparator | Any form of control group with no physical enrichment, including usual care or wait list control | Interventions compared two active intervention arms without a comparison/control group |
| Outcome | Children's physical (i.e., movement behaviors including total physical activity, intensity-specific physical activity and sedentary time, etc.), cognitive, psychological/affective, and/or social development | Other outcomes |
| Study design | Randomized controlled trials (RCT), cluster-RCT, non-randomized controlled trials | Systematic review, observational studies, and descriptive studies with no data |
| Publication type | Peer-reviewed journal articles, published in English | Conference abstracts, dissertations, books |
