# Supplementary material for: How can physical enrichment of school playgrounds improve movement behaviours and developmental outcomes in children and adolescents? A systematic review with meta-analysis
Source: Int J Behav Nutr Phys Act. 2025 Nov 22;22:161. doi: 10.1186/s12966-025-01856-y (PMC12751770; doi:10.1186/s12966-025-01856-y)
Supplement: Supplementary file 3 — Supplementary Material 3. [file 12966_2025_1856_MOESM3_ESM.docx]

Search strings for all databases

| **Search Terms** |
| --- |
| Set 1: Setting  school* or school-based or kindergarten or “early learning center” or childcare or “childcare center” or preschool or pre-school or college or “sport hall”  "Physical Education and Training"[Mesh] or "Child Care"[Mesh] or "Child Day Care Centers"[Mesh] |
| Set 2: Population  child* or adolescen* or youth or “young people” or teen* or student* or pupil* or infant or toddler  "Child"[Mesh] or "Adolescent"[Mesh] or "Infant"[Mesh] |
| Set 3: Intervention  S1 ((enrich or enrichment or enriching or modification or manipulation or “nature based” or upgrade or upgrading or rebuild or rebuilding or renovate or renovating or repurpose or repurposing or adapting or variation or variations or greening or greenness or marking) NEAR/4 (“physical structure” or equipment or material or environment or facility or facilities or outdoor or playground or schoolyard or “play area” or “play space” or playful))  S2 ((introduce or introducing or include or including or add or adding) NEAR/4 (nature or forest or aquatic or “water based” or water-based or “grass field” or “sport hall”))  S3 (change NEAR/4 (“play environment” or “playful environment” or “play-based environment” or “physical structure” or equipment or material or facility or facilities or outdoor or playground or schoolyard or “play area” or “play space” or playful))  "Environment, Controlled"[Mesh] or "Play and Playthings"[Mesh]  Set 3 S1 or S2 or S3 |
| **String** Set 1 AND Set 2 AND Set 4 (setting, population and intervention) |
|  |
| **String tailored to SportDiscuss and CINAHL** (EBSCOhost)  S1 school* or school-based or kindergarten or “early learning center” or childcare or “childcare center” or preschool or pre-school or college or “sport hall”  S2 child* or adolescen* or youth or “young people” or teen* or student* or pupil* or infant or toddler  S3 ((enrich or enrichment or enriching or modification or manipulation or “nature based” or upgrade or upgrading or rebuild or rebuilding or renovate or renovating or repurpose or repurposing or adapting or variation or variations or greening or greenness or marking) N4 (“physical structure” or equipment or material or environment or facility or facilities or outdoor or playground or schoolyard or “play area” or “play space” or playful))  S4 ((introduce or introducing or include or including or add or adding) N4 (nature or forest or aquatic or “water based” or water-based “grass field” or “sport hall”))  S5 (change N4 (“play environment” or “playful environment” or “play-based environment” or “physical structure” or equipment or material or facility or facilities or outdoor or playground or schoolyard or “play area” or “play space” or playful))  S6 S3 OR S4 OR S5  S7 S1 AND S2 AND S6 |
| **String tailored to Medline and APA PsycInfo (Ovid)**  #1 (school? or school-based or kindergarten or "early learning center" or childcare or "childcare center" or preschool or pre-school or college or "sport hall").ti. or (school? or school-based or kindergarten or "early learning center" or childcare or "childcare center" or preschool or pre-school or college or "sport hall").ab. or ("Physical Education and Training" or "Child Care" or "Child Day Care Centers").sh.  #2 (child? or adolescen? or youth or "young people" or teen? or student? or pupil? or infant or toddler).ti. or (child? or adolescen? or youth or "young people" or teen? or student? or pupil? or infant or toddler).ab. or ("Child" or "Adolescent" or "Infant").sh.  #3 ((enrich or enrichment or enriching or modification or manipulation or "nature based" or upgrade or upgrading or rebuild or rebuilding or renovate or renovating or repurpose or repurposing or adapting or variation or variations or greening or greenness or marking) adj4 ("physical structure" or equipment or material or environment or facility or facilities or outdoor or playground or schoolyard or "play area" or "play space" or playful)).ti. or ((enrich or enrichment or enriching or modification or manipulation or "nature based" or upgrade or upgrading or rebuild or rebuilding or renovate or renovating or repurpose or repurposing or adapting or variation or variations or greening or greenness or marking) adj4 ("physical structure" or equipment or material or environment or facility or facilities or outdoor or playground or schoolyard or "play area" or "play space" or playful)).ab.  #4 ((introduce or introducing or include or including or adding) adj4 (nature or forest or aquatic or "water based" or "water-based" or "grass field" or "sport hall")).ti. or ((introduce or introducing or include or including or adding) adj4 (nature or forest or aquatic or "water based" or "water-based" or "grass field" or "sport hall")).ab.  #5 (change adj4 ("play environment" or "playful environment" or "play-based environment" or "physical structure" or equipment or material or facility or facilities or outdoor or playground or schoolyard or "play area" or "play space" or playful)).ti. or (change adj4 ("play environment" or "playful environment" or "play-based environment" or "physical structure" or equipment or material or facility or facilities or outdoor or playground or schoolyard or "play area" or "play space" or playful)).ab.  #6 ("Environment, Controlled" or "Play and Playthings").sh.  #7 #3 or #4 or #5 or #6  #8 #1 AND #2 AND #7 |
| **String tailored to Web of Science**  #1 TS=(school* or school-based or kindergarten or “early learning center” or childcare or “childcare center” or preschool or pre-school or college)  #2 TS=(child* or adolescen* or youth or “young people” or teen* or student* or pupil* or infant or toddler)  #3 TS=((enrich or enrichment or enriching or modification or manipulation or “nature based” or upgrade or upgrading or rebuild or rebuilding or renovate or renovating or repurpose or repurposing or adapting or variation or variations or greening or greenness or marking) NEAR/4 (“physical structure” or equipment or material or environment or facility or facilities or outdoor or playground or schoolyard or “play area” or “play space” or playful))  #4 TS=((introduce or introducing or include or including or add or adding) NEAR/4 (nature or forest or aquatic or “water based” or water-based or “grass field” or “sport hall”))  #5 TS=(Change NEAR/4 (“play environment” or “playful environment” or “play-based environment” or “physical structure” or equipment or material or facility or facilities or outdoor or playground or schoolyard or “play area” or “play space” or playful))  #6 #3 OR #4 OR #5  #7 #1 AND #2 AND #6 |
| **String tailored to Scopus**  #1 TITLE-ABS (school* OR school-based OR kindergarten OR "early learning center" OR childcare OR "childcare center" OR preschool OR pre-school OR college OR "sport hall")  #2 TITLE-ABS (child* OR adolescen* OR youth OR "young people" OR teen* OR student* OR pupil* OR infant OR toddler)  #3 TITLE-ABS ((enrich OR enrichment OR enriching OR modification OR manipulation OR "nature based" OR upgrade OR upgrading OR rebuild OR rebuilding OR renovate OR renovating OR repurpose OR repurposing OR adapting OR variation OR variations OR greening OR greenness OR marking) W/4 ("physical structure" OR equipment OR material OR environment OR facility OR facilities OR outdoor OR playground OR schoolyard OR "play area" OR "play space" OR playful))  #4 TITLE-ABS ((introduce OR introducing OR include OR including OR add OR adding) W/4 (nature OR forest OR aquatic OR "water based" OR water-based OR “grass field” or “sport hall”))  #5 TITLE-ABS (change W/4 ("play environment" OR "playful environment" OR "play-based environment" OR "physical structure" OR equipment OR material OR facility OR facilities OR outdoor OR playground OR schoolyard OR "play area" OR "play space" OR playful))  #6 #3 or #4 or #5  #7 #1 AND #2 AND #6 |
| **String tailored to Cochrane Library**  #1 (school* or school-based or kindergarten or “early learning center” or childcare or “childcare center” or preschool or pre-school or college or “sport hall”):ti,ab,kw  #2 (child* or adolescen* or youth or “young people” or teen* or student* or pupil* or infant or toddler):ti,ab,kw  #3 ((enrich or enrichment or enriching or modification or manipulation or “nature based” or upgrade or upgrading or rebuild or rebuilding or renovate or renovating or repurpose or repurposing or adapting or variation or variations or greening or greenness or marking) near/4 (“physical structure” or equipment or material or environment or facility or facilities or outdoor or playground or schoolyard or “play area” or “play space” or playful))  #4 ((introduce or introducing or include or including or add or adding) near/4 (nature or forest or aquatic or “water based” or water-based “grass field” or “sport hall”))  #5 (change near/4 (“play environment” or “playful environment” or “play-based environment” or “physical structure” or equipment or material or facility or facilities or outdoor or playground or schoolyard or “play area” or “play space” or playful))  #6 #3 or #4 or #5  #7 #1 and #2 and #6 |
