# Supplementary material for: How can physical enrichment of school playgrounds improve movement behaviours and developmental outcomes in children and adolescents? A systematic review with meta-analysis
Source: Int J Behav Nutr Phys Act. 2025 Nov 22;22:161. doi: 10.1186/s12966-025-01856-y (PMC12751770; doi:10.1186/s12966-025-01856-y)
Supplement: Supplementary file 4 — Supplementary Material 4. [file 12966_2025_1856_MOESM4_ESM.docx]

Baquet et al., 2018: 15000 euro per school

Blaes et al., 2013: 15000 euro per school

Cardon et al., 2009: 250 euro per school for equipment; markings painted by researchers

Christiansen et al., 2017: 40000-300000 for renovation

Crust et al., 2014: not defined

Engelen et al., 2013; Bundy et al., 2017: recycled material

Farmer et al., 2017: initial grant of 15000 NZD, but strategies with no cost

Hamer et al., 2017: not defined

Huberty et al., 2014: not defined

Hyndman et al., 2014: recycled material

Janssen et al., 2015: not defined

Kelly et al., 2012: not defined

Kelz et al., 2015: not defined

López-Fernández et al., 2016: not defined

Loucaides et al., 2009: not defined

Ng et al., 2020: not defined

Nigg et al., 2019: not defined

Ridgers et al., 2010: 20000 pounds per school

Sanz-Mas et al., 2025: not defined

Stratton 2000; Stratton & Leonard, 2002: not defined

Stratton & Mullan, 2005: painted probably by researchers or schools

Tucker et al., 2017: not defined

Verstraete et al., 2006: not defined

Van Dijk-Wesselius et al., 2018: not defined
