# Supplementary material for: How can physical enrichment of school playgrounds improve movement behaviours and developmental outcomes in children and adolescents? A systematic review with meta-analysis
Source: Int J Behav Nutr Phys Act. 2025 Nov 22;22:161. doi: 10.1186/s12966-025-01856-y (PMC12751770; doi:10.1186/s12966-025-01856-y)
Supplement: Supplementary file 5 — Supplementary Material 5. [file 12966_2025_1856_MOESM5_ESM.docx]

Measures of heterogeneity for the meta-analysis of the movement behaviour outcomes

| **Outcome** | **I^2^ (%)** | **Tau^2^ (g unit)** | **Tau (g unit)** |
| --- | --- | --- | --- |
| SED | 74 | 0.04 | 0.19 |
| LPA | 43 | 0.01 | 0.12 |
| MPA | 90 | 0.09 | 0.31 |
| VPA | 34 | 0.01 | 0.08 |
| MVPA | 82 | 0.06 | 0.24 |
| Steps per minute | 95 | 0.45 | 0.67 |
