# Supplementary material for: How can physical enrichment of school playgrounds improve movement behaviours and developmental outcomes in children and adolescents? A systematic review with meta-analysis
Source: Int J Behav Nutr Phys Act. 2025 Nov 22;22:161. doi: 10.1186/s12966-025-01856-y (PMC12751770; doi:10.1186/s12966-025-01856-y)
Supplement: Supplementary file 6 — Supplementary Material 6. [file 12966_2025_1856_MOESM6_ESM.docx]

GRADE rating of quality of evidence for SED, LPA, MPA, VPA, MVPA, and number of steps per minute

| **GRADE criteria** | **Rating** | **Comments** | **Quality of evidence** |  |
| --- | --- | --- | --- | --- |
| *Outcome: Physical Activity profile* | | | | |
| Study design | High |  | **Low** |  |
| Risk of Bias | Medium (-1) | High risk of bias some concerns in critical domains |  |  |
| Inconsistency | No |  |  |  |
| Indirectness | No |  |  |  |
| Imprecision | Low (-1) | Wide Confidence Intervals |  |  |
| Publication bias | Undetected |  |  |  |
| Other (upgrading factors) | No |  |  |  |
