# Supplementary material for: How can physical enrichment of school playgrounds improve movement behaviours and developmental outcomes in children and adolescents? A systematic review with meta-analysis
Source: Int J Behav Nutr Phys Act. 2025 Nov 22;22:161. doi: 10.1186/s12966-025-01856-y (PMC12751770; doi:10.1186/s12966-025-01856-y)
Supplement: Supplementary file 8 — Supplementary Material 8. [file 12966_2025_1856_MOESM8_ESM.docx]

**Outcome: VPA**

**Outcome: MVPA**
