# Supplementary material for: How can physical enrichment of school playgrounds improve movement behaviours and developmental outcomes in children and adolescents? A systematic review with meta-analysis
Source: Int J Behav Nutr Phys Act. 2025 Nov 22;22:161. doi: 10.1186/s12966-025-01856-y (PMC12751770; doi:10.1186/s12966-025-01856-y)
Supplement: Supplementary file 9 — Supplementary Material 9. [file 12966_2025_1856_MOESM9_ESM.docx]

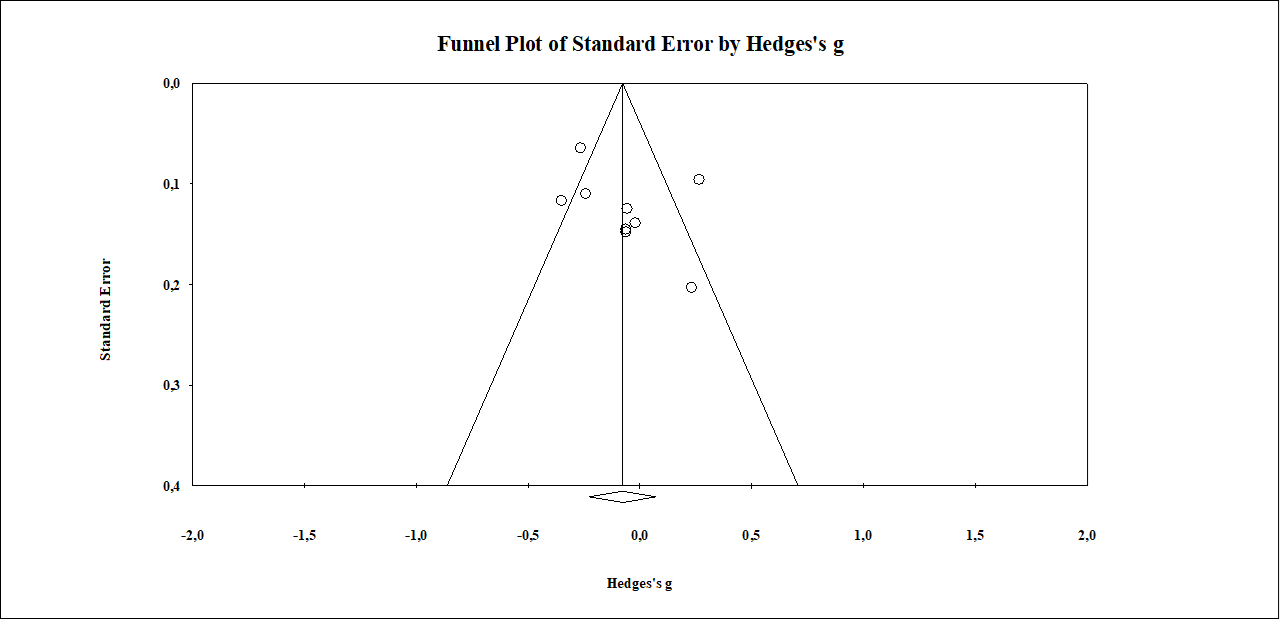


Figure 1 Funnel plot for the publication bias of SED

Figure 2 Funnel plot for the publication bias of LPA

Figure 3 Funnel plot for the publication bias of MPA

Figure 4 Funnel plot for the publication bias of VPA


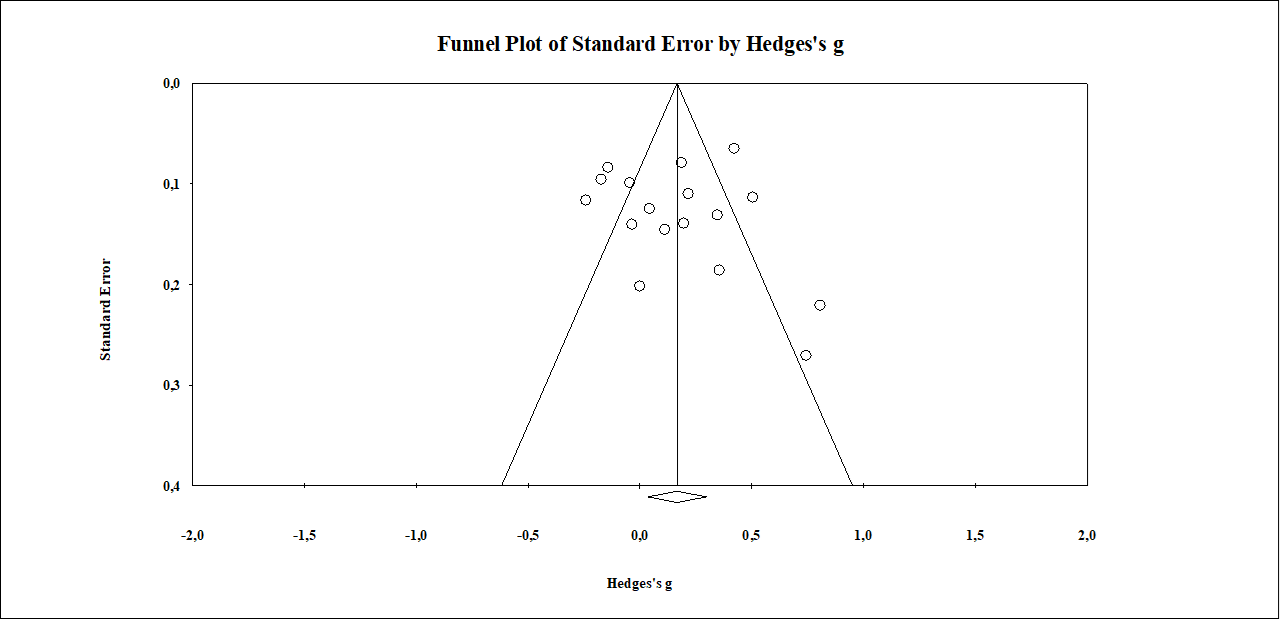


Figure 5 Funnel plot for the publication bias of MVPA

Figure 6 Funnel plot for the publication bias of the number of steps per minute
